# Sustainable Stabilization of Silty Sand Using Recycled Industrial Polymer Reinforcement with a Hybrid Lime–Cement Binder

**DOI:** 10.3390/polym18101264

**Published:** 2026-05-21

**Authors:** Ayad Lounas, Yazeed A. Alsharedah, Sadek Deboucha, Yasser Altowaijri

**Affiliations:** 1 InfraRES Laboratory, Department of Civil Engineering, Faculty of Sciences and Technology, University of Souk Ahras, Souk Ahras 41000, Souk Ahras, Algeria; l.ayad@univ-soukahras.dz; 2 Department of Civil Engineering, College of Engineering, Qassim University, Buraydah 52541, Qassim, Saudi Arabia; y.alsharredah@qu.edu.sa; 3 Department of Civil Engineering, University Mohamed El Bachir El Ibrahimi of Bordj Bou Arreridj, Bordj Bou Arreridj 34000, Bordj Bou Arreridj, Algeria

**Keywords:** sustainability, stabilization, subgrade layer, silty sand, industrial waste plastic, lime, cement, combination effects

## Abstract

Stabilizing weak soils is a well-known pavement and geotechnical engineering technique. This technique involves introducing minimal cementitious materials to improve the soil’s geotechnical characteristics. This paper investigates the use of recycled industrial polymer waste (IPW) as a reinforcement material in the presence of cementitious binders to stabilize weak silty sand soil (SM), supporting sustainable engineering practices. The randomly distributed IPW were added as percentages of 0%, 5%, and 10% to a mixture of lime soil and cement soil, with varying amounts of 0% to 6% of lime (L) and 0% to 6% of ordinary Portland cement (OPC), respectively. The laboratory experiments were conducted on natural and stabilized samples in wet (unsoaked) and submerged (soaked) conditions. The experimental program included Proctor compaction, California bearing ratio (CBR), unconfined compressive strength (UCS), durability tests, scanning electron microscopy (SEM), energy dispersive spectroscopy (EDS), and X-ray diffraction analyses. The resilient modulus (Mr) was estimated using an empirical equation. The outcomes of this experimental study show that adding a combination of IPW shreds with a small amount of L and/or OPC to the SM soil provides a significant increase in the UCS, CBR, durability and Mr values compared with case of SM with only L, which allows for superior characteristics and increases strength and stiffness parameters throughout any phase of earthwork construction design, resulting in stronger and stiffer subgrades. These results were reinforced by microstructural observations from SEM, EDS, and DRX, confirming the formation of cementitious gels and chemical compounds, consistent with the macro-scale mechanical improvements. The expected practical outcomes include potential reductions in pavement thickness, which can help lower pavement stabilization costs and extend its service life. Additionally, the use of waste materials to replace raw materials contributes to decreased energy consumption and emissions, although detailed assessments are needed to quantify these effects.

## 1. Introduction

Transported soils can present several construction-engineering issues, especially when used in transportation engineering projects. Since transportation networks often span large geographic areas and require substantial material quantities, sourcing high-quality foundation soils for subgrades and subbases is rarely feasible. As a result, contractors commonly rely on locally available soils that meet AASHTO or equivalent local standards. Among these soils, aeolian sands and silty sand—characterized by rounded grains, fine particle sizes, and mixed sand–silt composition—are widely recognized as problematic. These deposits, occurring across regions such as the southern United States, eastern Saudi Arabia, and northern Africa, often exhibit undesirable behavior under varying moisture and loading conditions, making them unsuitable for direct use in pavement subgrades [1]. Ensuring an adequate subgrade layer is essential to provide adequate road performance, as weakened subgrade soils can lead to premature pavement distress and costly maintenance [2,3]. Over the last five decades, the field of geotechnical engineering has seen increased use of soil improvement techniques, particularly in pavement engineering. These techniques include mechanical compaction (i.e., dynamic compaction, static compaction, nonbinding additives), chemical (by adding cementitious additives to the original soil, such as Portland cement, lime, calcium chloride, fly ash, blast furnace slag, steel slag, phosphogypsum, and other additives [4,5,6,7,8,9], rigid inclusions (such as piles, sand columns, stone columns, deep soil mixing (DSM), etc.) and hydraulic improvement (assisted consolidation using PVD or preloading) or a combination of the above.

For instance, Koukouzas et al. [8] carried out an exhaustive literature assessment of diverse soil-stabilizing techniques; they concluded that fly ash has a high potential to promote self-cementing plus soil stability. Fly ash is usually mixed with small amounts of cement to stabilize soil [7]. The reported mixture percentages range between 5 and 20% of the dry weight of the soil sample. For decades, Lime and cement have been used to stabilize weak subgrade soils [10,11,12]. Both materials have exceptional aptitudes to interrelate with a number of soils. To stabilize medium- to high-plasticity soils, lime is preferred as an additive, bringing beneficial changes to the engineering characteristics of soils. Lime stabilizes by cation exchange, flocculation, agglomeration, lime carbonation, and pozzolanic processes. These processes are time-bound, producing phase modification changes in soil properties, with pozzolanic reactions continuing over time. These pozzolanic reactions include interactions between soil silica and/or alumina and lime to produce various cementation products, enhancing soil strength [13]. Reference [14] studied the performance of clay stabilized soil with variable amounts of lime (4 and 12% by weight) for short and long-term conditions. The specimens were cured in a wet environment for one month and ten years. The results indicated that the compression strength of the stabilized specimens grew around 8-fold over 1 month, continuing to increase to approximately 21-fold after 10 years. However, increased lime concentrations may lead to the formation of silica gel, a porous substance that decreases and diminishes the soil skeleton’s strength [11,15]. A combination of lime and cement was used by many (e.g., [16,17,18] to treat a wider variety of silty sand and clayey soils, and the findings showed that cement outperformed lime stabilization in terms of durability. While this cement stabilization technique results in improved bearing capacity and stiffness properties, this technique is financially expensive and environmentally polluting, encouraging industries and researchers to investigate alternatives, such as by-products or waste materials, to reduce the use of these materials, which can be used alone or combined with traditional agents for soil improvement [19,20].

Due to its low cost and environmental benefits, plastic waste has been a subject of extensive research in recent years as a new soil amendment material. The recycling of plastic waste for geotechnical applications can substantially decrease the amount of waste disposed of and the amount of greenhouse gas emitted. The use of plastic waste in soil stabilization is thus a sustainable and environmentally friendly practice that contributes to cleaner production and sustainable development. Many studies have been conducted on the behavior of soils reinforced with various types of plastic waste fibers, such as general waste plastics [21,22,23,24], polypropylene fibers [25,26], and polyethylene terephthalate (PET) fibers [27,28]. Past studies have also demonstrated that the addition of plastic waste can substantially decrease desiccation cracking in soils that are repeatedly wetted and dried, and thus enhance the overall stability and durability of soil-based infrastructure [29,30]. Special focus has been placed on the use of PET waste in combination with cementitious stabilizers to improve the properties of the soil [31]. Olutaiwo and Ezegbunem [32] found that the soil density and California Bearing Ratio (CBR) were significantly enhanced by the mixture with 7% cement and 10% PET strips. Likewise, Ziani et al. [33] reported significant improvements in unconfined compressive strength (UCS) and CBR with the addition of recycled PET fibers (0–10%) and ordinary Portland cement (OPC) (0–4%). Urian et al. [34] also showed that adding small amounts of PET waste has a positive effect on the shear strength and compressibility properties of the soil. Based on machine learning analysis, Vahedi and Koohmishin [35] concluded that PET content is one of the main parameters affecting the strength behavior of PET–lime-stabilized clay. Furthermore, Silveira et al. [36] reported that the addition of PET to the cement-treated silty sand enhanced the strength and durability. Similar results were obtained by El Majid et al. [37], who demonstrated that recycled polypropylene fibers improved soil ductility and strength and promoted the principles of the circular economy. Attom et al. [31] also showed that the incorporation of 6–8% plastic–cement mixtures in expansive clay resulted in significant improvement in engineering properties, such as reduction in swelling pressure, swell potential, compressibility, and compression index after curing. The results of these studies collectively indicate the potential of plastic waste as an effective and sustainable geotechnical stabilization material. Although the research is increasing, few studies have examined the simultaneous effects of recycled industrial waste plastic (IPW) shreds, lime and cement on the stabilization of silty sand soils. This is crucial for sustainable construction and minimizing the use of virgin aggregates and binders with high carbon emissions. Based on this, the present study investigates the feasibility of using IPW shreds as a reinforcing material for silty sand subgrade stabilization along with lime and cement. A detailed laboratory-testing program was carried out to assess the impact of these additives on compaction properties, unconfined compressive strength, California Bearing Ratio, durability, estimated resilient modulus, scanning electron microscopy (SEM), energy-dispersive spectroscopy (EDS), and X-ray diffraction (XRD) analyses. The results will be used to develop environmentally friendly and mechanically efficient stabilization methods for transportation infrastructure applications.

## 2. Materials and Methods

### 2.1. Materials

This section describes the natural soil, industrial waste plastic, lime, and cement employed in the current experiment.

#### 2.1.1. Soil

**Figure 1:**
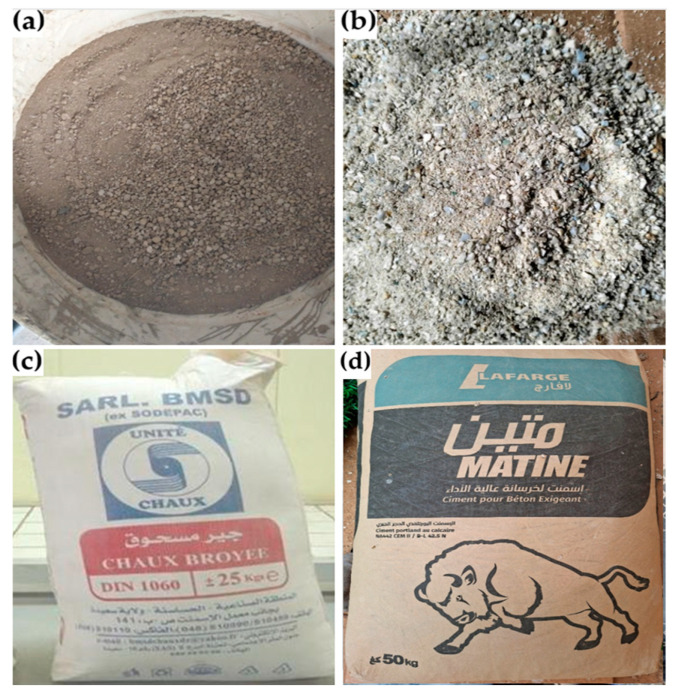
Materials: (**a**) Natural soil sample, (**b**) Industrial waste plastic shreds, (**c**) Lime, (**d**) Cement.

A natural soil sample was taken from the road construction site in Bordj Bou Arreridj (BBA), Algeria. All experiments were performed in the laboratory of the civil engineering department at the University of BBA. The identification tests of soil were performed using the applicable ASTM standards, including SEM, Proctor compaction, UCS, and CBR tests. Table 1 and Table 2 present the physical parameters and chemical components of the natural soil, respectively. Figure 1 depicts material samples used in the inquiry. This soil is classed as well-graded sand with moderate plasticity particles (A-2-4) using the AASHTO, 1982 [38] classification standard. These classifications are typical for silty sands with moderate fines and low to moderate plasticity. According to the Unified Soil Classification System (ASTM D2487-11) [39], it was classed as silty sand with moderate plasticity. Soils in this category typically have poor engineering properties and must be stabilized when exposed to external stresses. Furthermore, the silty concentration may cause shrinkage and swelling, though not as much as in exclusively clayey soils. It is more susceptible to moisture changes, which might affect its strength or stiffness. The silt fraction affects soil stability, particularly during loading or changing moisture conditions.

#### 2.1.2. Industrial Waste Plastic (IPW)

The IPW used in this study is an industrial waste plastic that came from a recycled admixture of polyethylene terephthalate (PET) and high-density polyethylene (HDPE). The IPW shreds are irregular in shape, resulting from mechanical industrial cutting. The particles exhibit a fibrous to flaky morphology with rough and uneven edges, which enhances mechanical interlocking with the soil matrix. The particle size typically ranges from approximately 3 to 15 mm in length and 1 to 3 mm in width; the material is generally transparent to translucent, with particles appearing in mixed colors such as light white, blue, green, and clear (Figure 1b), the PET is the most commonly used thermoplastic, which is a polycondensation product of terephthalic acid (C8H6O4) with ethylene glycol (C_2_H_6_O_2_). This transparent polyester, with its good stability and mechanical and chemical properties, is lightweight and highly inert [40,41]. This allows its use in the food sector. However, the stability of recycled PET is currently a topic of debate, with some arguing that products made from recycled PET are of lower quality [42]. Their grinding also results in the production of many flakes (PET). It is a far less costly alternative than other chemicals used to improve the CBR, compaction, shear strength, and bearing capacity overall, such as cement and lime. However, given its light weight, cheap cost and scalability, PET is considered to have potential as a successful soil additive for enhancing and strengthening its geotechnical properties. Its remarkable attributes include exceptional wear resistance, a low friction coefficient, and a high flexural modulus [43]. According to [44], comparative research on the impact of different waste plastic products on the engineering properties of clay soil, the CBR and UCS values were higher with PE admixture than with polypropylene (PP) admixture. Considering that, PE offers a higher tensile strength compared to PP and PVC (polyvinyl chloride), it can be employed as a supplement for achieving the best results. Since IPW shreds have a mostly smooth surface, this can reduce the bonding and adhesion between shreds and soil particles or other material additions. We prefer to propose this study to use the IPW shreds, to increase the likelihood of obtaining more positive results. As far as we know, there are no previous studies on the use of IPW shreds with both lime and cement in strengthening silty sand soils.

#### 2.1.3. Lime

The quicklime utilized in this investigation was produced by BMSD-SARL in Saïda, Algeria (southwest of the national territory). Table 3 presents the characteristics of this quicklime, which have already been given on the container of the lime, offering a comprehensive overview of its characteristics.

#### 2.1.4. Cement

The hydraulic binder used in our study is MATINE cement CEM II/B-L 42.5 N (NA 442); gray Ordinary Portland Cement (OPC) for high-performance concrete intended to construct civil engineering structures, infrastructure, and building superstructures. Table 4 shows the physical properties and chemical composition of MATINE cement. According to the cement container provided by the producing industry, which is certified and is in compliance with Algerian (NA 442-2013) [45] and European (EN 197-1) [46] standards.

### 2.2. Methodology and Scope of Work

This study established an experimental plan to evaluate the effects of combining industrial waste plastic (IPW) shreds with cement and lime stabilizers for strengthening silty sand soil. Native and stabilized soil samples were prepared by oven-drying natural silty sand soil at 105 degrees Celsius for 24 h. Mixtures were then formulated with varying ratios of IPW (0%, 5%, and 10%), lime (0%, 2%, 4%, and 6%), and cement (0%, 2%, 4%, and 6%) by total soil weight, as detailed in Table 5. The selected concentration ranges for IPW, lime, and cement were determined through a combination of literature review, practical considerations, and preliminary experimental investigations. The waste plastic content range was informed by previous research, which indicated that lower percentages are generally sufficient to enhance soil behavior without causing poor workability or material segregation [33,34,35,36]. Accordingly, a range of 0–10% was adopted to capture the material’s effect. The lime and cement ranges align with values commonly reported in prior studies on soil stabilization, where typical contents fall within 0–10% for both additives [13,17,31,48]. These ranges have demonstrated improvements in mechanical properties while remaining economically and practically feasible. Preliminary tests further refined these ranges to ensure they captured both the lower and upper bounds of effective performance. Initial trials indicated that values outside the selected intervals produced negligible improvement or diminishing returns. The chosen ranges were intended to balance performance optimization, material efficiency, and consistency with existing literature.

#### 2.2.1. Sample Preparation

**Figure 2:**
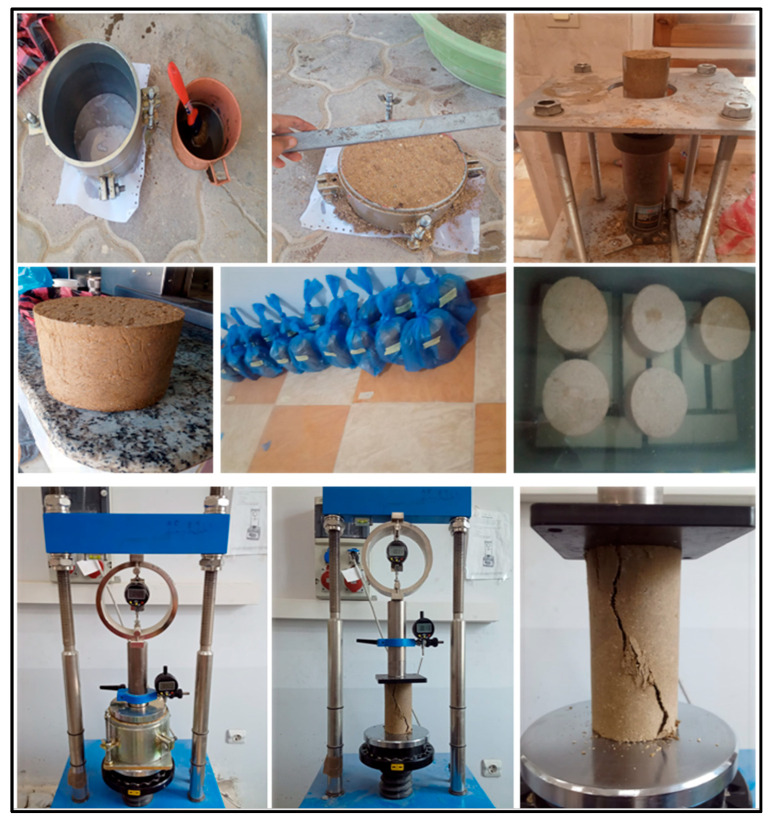
The laboratory experimental steps of the current research.

A total of nineteen (19) mixtures were prepared, with three specimens produced for each composition. This section outlines the sample preparation procedure used for all tested materials. Due to the differences in density between soil, IPW shreds, lime, and cement, a dry mixing approach was adopted to ensure homogeneous distribution. Initially, the dry soil was thoroughly mixed with lime and/or cement. Water was then gradually added to reach the optimum moisture content (OMC) of each mixture, ensuring uniformity and preventing the formation of agglomerates. Subsequently, the IPW shreds were incorporated and mixed until a consistent blend was achieved. Standard Proctor compaction tests were conducted to determine the OMC of mixtures containing IPW, lime, and cement, in accordance with ASTM D698 [49]. The obtained OMC values were then used for the preparation of specimens for California Bearing Ratio (CBR), Unconfined Compressive Strength (UCS), and durability tests. To ensure the specimens’ dry density, they were homogeneously compressed in five layers in the mold. Some samples were placed under a hydraulic machine to apply an additional load expected at 1–2 kPa, resulting in a more uniform and even mixture of the different elements. To facilitate counting and writing down the samples, we name each sample by its code: natural silty sand soil (Native), stabilized samples (IPW (P%) lime (L%) cement (C%)). The samples were subsequently covered using a plastic film to maintain water evaporation to avoid moisture loss until testing day; some steps of preparation, retention, and testing samples are presented in Figure 2.

#### 2.2.2. Scanning Microscope (SEM)

SEM tests were conducted on a selected sample of untreated and treated soils. The SEM analysis is a type of microstructure imaging technology that takes advantage of the properties of the sample surface materials. The SEM (JEOL JSM-7610FPlus FESEM) was adopted for scanning electron microscopy. The samples were dried in the oven at a temperature of 60 °C and then cut into 10 mm × 10 mm × 10 mm cubes with a blade. During testing, the surface was smoothed, and a relatively flat, fresh surface was exposed by manual opening; this fresh surface adhered to the bracket with double-sided adhesive. After the above test preparation process is completed, the SEM image acquisition in high vacuum mode can be carried out. The magnifications of the images collected in this paper are all 35 to 150 times.

#### 2.2.3. X-Ray Diffraction (XRD)

XRD analysis is a kind of technology that utilizes the diffraction effect of X-rays in crystal materials to analyze the structure of materials. It can be applied to the qualitative and semi-quantitative analysis of crystal phases. The X-ray diffractometer (Empyrean PANalytical) was used for phase diffraction analysis. The Empyrean PANalytical 4 kW XRD system is equipped with a copper anode material and generator settings of 40 mA and 40 kV. The ratio of K-Alpha2 (Å) to K-Alpha1 (Å) was 0.5, and that of K-Beta (Å) was 1.39225. The specimen length was 10 mm, and the measurement was taken at 25 °C. The Gonio scan axis has the start and end positions of 10.000 and 88.820° (2θ), a step size of 0.0200° (2θ), and a scan time of 60 min. High Score Plus V4.8 with Rietveld phase identification and crystal composition was used for the analysis.

## 3. Experimental Results and Discussion

### 3.1. Effect of the Combination of IPW with Lime and Cement on the Compaction Characteristics

**Figure 3:**
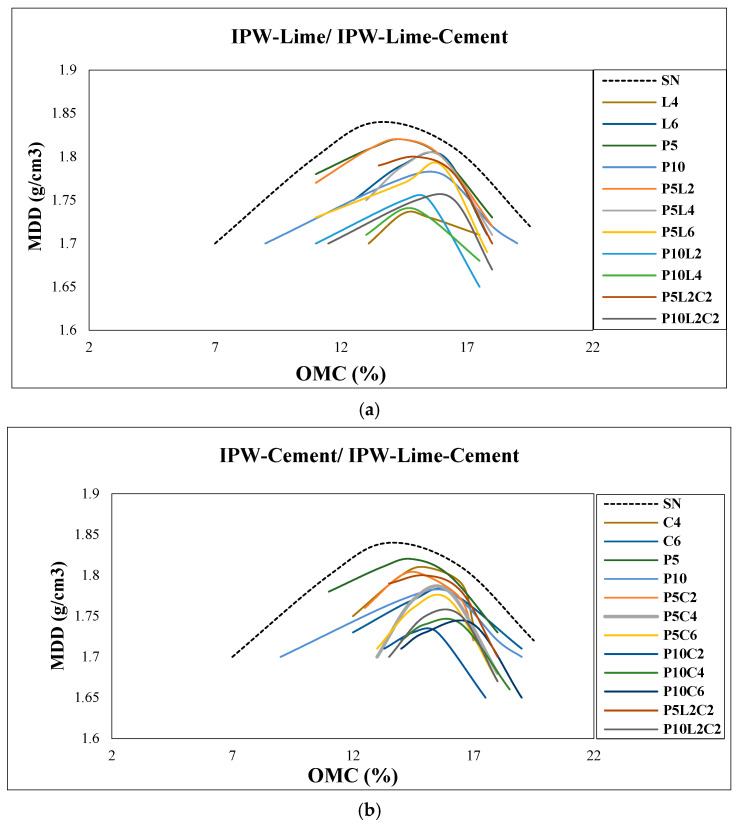
Effect of IPW and binder on MDD and OMC for different mixtures: (**a**) IPW-Lime/IPW-Lime-Cement, (**b**) IPW-Cement/IPW-Lime-Cement.

Figure 3a,b shows the outcomes of the MDD and OMC for the native and stabilized silty sand soil samples using combined mixtures of IPW-lime and IPW-cement, respectively, as stabilizers at different content percentages. For the native soil, the OMC value was 14%, and the MDD value was 1.835 g/cm^3^.

As can be seen in the compaction test results, the MDD decreases for all coupled IPW-lime, IPW-cement, and IPW-lime-cement stabilized soil samples compared to the native soil sample. The MDD of stabilized soil samples decreases as the IPW content increases, as can be noticed as both lime and cement contents increase. The maximum value of MDD is observed firstly for both the mixture of 5% IPW combined with 2% lime or 2% cement, separately; this can be attributed to the low quantities of the IPW and both lime and cement in the mixture. After that, it decreases as the IPW, lime, and cement contents increase, with samples with 10% IPW and 6% lime or cement producing the lowest MDD value. The MDD of the samples decreases as the plastic content increases because the plastic IPW shreds are a much lighter material than the soil particles. Furthermore, the presence of IPW shreds creates a barrier-like effect and prevents the rearrangement of the soil particles, which in turn decreases the overall density of the compacted stabilized soil sample.

The OMC values increase and move to the right with an increase in the percentage of IPW with lime and with cement as mixtures. Given that, IPW is an inert material; the increase in moisture content could be caused by the absorption of a part of the water by lime, the cement, or both. A sample with 10% IPW and 4% lime or 6% cement produced the highest OMC values of 16.7%. This is due to the sensitivity to water being due to the hydraulic aspect of the lime and cement, and the nature of the silty sand soil. On the other hand, the bipolar water molecules adhere to IPW shreds and form a film around them, and as the IPW contents increase, the cumulative surface area and size of the film increase, causing an increase in OMC values. Furthermore, as can be noticed, both lime and cement contents show a similar effect on both MDD and OMC; despite the physical and chemical composition of both, this similar effect is due to the smaller amounts of both used in mixtures and the predominance of the IPW shreds effect.

### 3.2. Effect of the Combination of IPW with Lime and Cement on the Californian Bearing Ratio CBR

**Figure 4:**
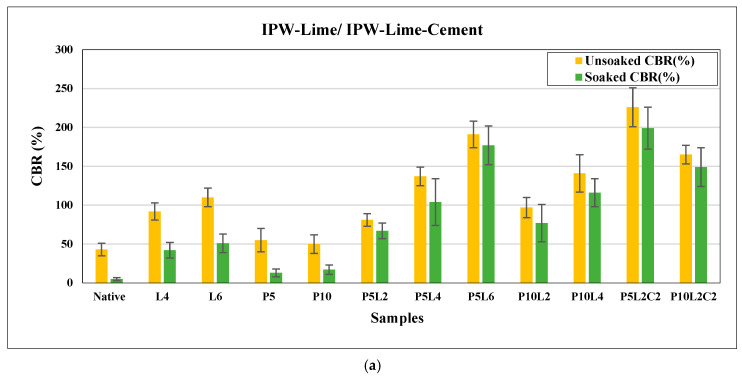

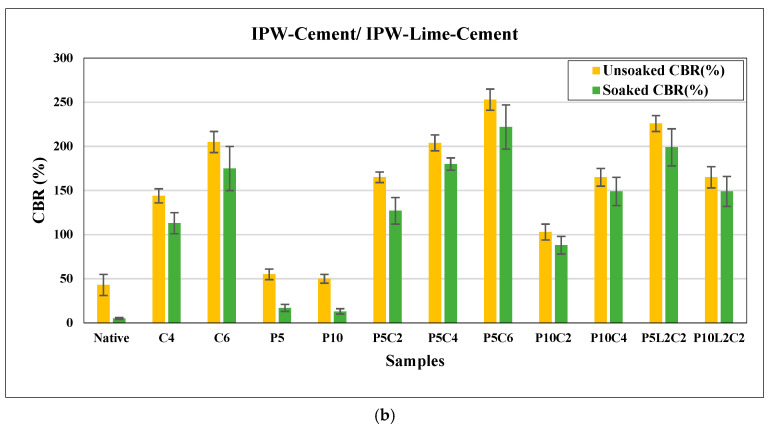
Effect of IPW and binders on CBR results for both conditions (soaked and unsoaked) for different mixtures: (**a**) IPW-Lime/IPW-Lime-Cement, (**b**) IPW-Cement/IPW-Lime-Cement.

The CBR is the most widely accepted parameter for pavement design. This investigation evaluated unsoaked and soaked conditions to evaluate the effect of IPW combined with lime and/or cement on CBR values for this silty sand soil. The CBR values for native and stabilized soil samples in unsoaked and soaked conditions at 28 days are presented in Figure 4. The result showed that an increase in the ratio of IPW, lime, and cement results in a significant improvement.

The CBR tests for both native and stabilized silty sand soil samples were conducted according to ASTM D1883 [50] procedures. Soaked and unsoaked conditions were examined in CBR, and the results are presented in Figure 4a,b. The findings indicate that combining waste plastic IPW shreds, lime, and cement increases the CBR results for both conditions, indicating an improvement in soil bearing capacity.

In Figure 4a, the CBR results of the native soil sample stabilized IPW-lime samples, and stabilized IPW-lime-cement samples are presented. The CBR result of the native silty sand mixture was 43% and 2% under unsoaked and soaked conditions, respectively. The addition of 5% IPW and 2% lime enhances the values of CBR for different mixtures. The best CBR value was observed in the P5L6 (5% IPW + 6% lime) mixture, displaying 30 times and 3 times increases in CBR values when the soil was stabilized with IPW and binder under soaked and unsoaked conditions, respectively. In addition, the addition of 5% IPW with 2% lime and 2% cement, as presented in P5L2C2, increased CBR values by about 90% and 110% for both conditions, soaked and unsoaked, respectively. Furthermore, the addition of 10% IPW with 2% lime and 2% cement improved CBR values by about 120% and 152% under both curing conditions for the mixture of P10L2C2 (10% IPW + 2% lime + 2% cement), displaying 60 times and 3.53 times increase compared to the CBR of native soil under soaked and unsoaked conditions. It has been observed that this presents the most significant enhancement for both soaked and unsoaked conditions. The amelioration of CBR results under both curing conditions indicates a growing bearing capacity of the soil.

In Figure 4b, the CBR results of soil-stabilized samples with IPW-lime-cement compared to the native sample and stabilized IPW cement samples are presented. Through the addition of IPW shreds of 5% and 10%, combined with cement by 2%, 4%, and 6%, the CBR values increased under soaked and unsoaked conditions. It indicated a huge enhancement in the CBR values compared to the results of the IPW-lime combination at the same percentages of mixtures. This enhancement demonstrates the effect of cement bonding and the time required for hardening, which gives the samples relatively strong bonds compared to the lime ones. It has been observed through various ratios of IPW and cement that the CBR values increased under both curing conditions. This increase in CBR is a function of both IPW and cement contents. The highest CBR values were observed in the P10C4 sample (10% IPW + 4% cement), reaching 107% and 151% under soaked and unsoaked conditions, and that is relatively close to the P10L2C2 (10% IPW + 2% lime + 2% cement) CBR values of 120% and 152% for soaked and unsoaked conditions. This result supports the idea that mixing lime with cement is better than using lime or cement separately for both short and long terms. On the other hand, it has been observed that less enhancement was in the P10C2 sample (10% IPW + 2% cement), reaching 52% and 74% for soaked and unsoaked conditions. This can be demonstrated by the predominance of IPW shreds with a lack of cement or lime bonds, the same observation on the P10L2 sample CBR values presented in Figure 4a.

### 3.3. Effect of the Combination of IPW with Lime and Cement on the Unconfined Compressive Strength (UCS)

**Figure 5:**
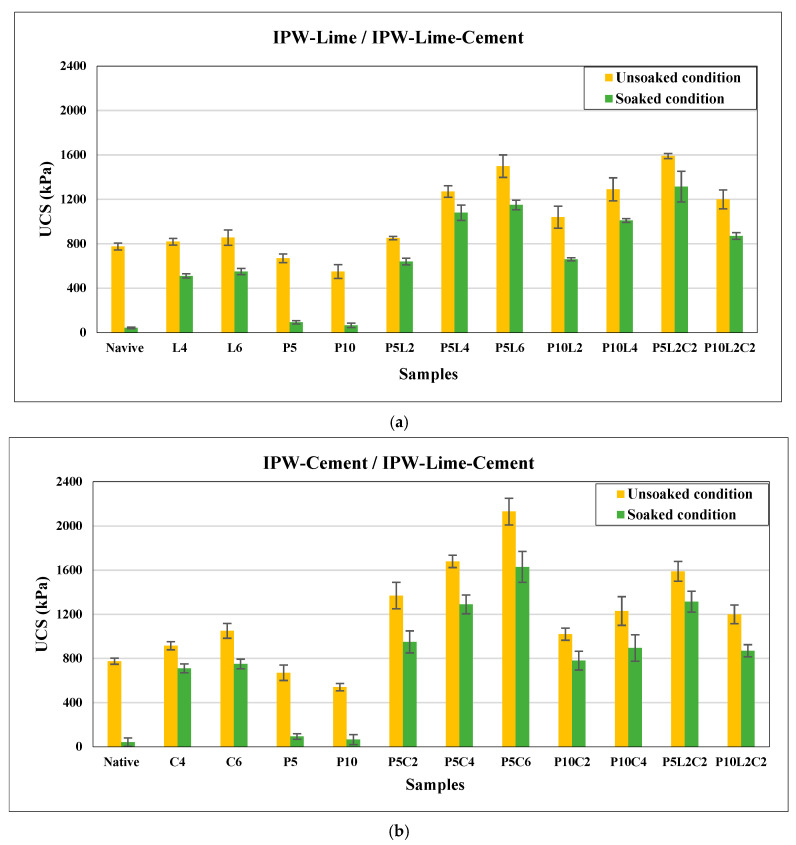
Effect of IPW and binder on UCS results for both conditions (soaked and unsoaked) for different mixtures: (**a**) IPW-Lime/IPW-Lime-Cement, (**b**) IPW-Cement/IPW-Lime-Cement.

In Figure 5, the UCS results of native and stabilized silty sand soil samples in both curing conditions at 28 days are presented. The findings indicate that combining IPW shreds with lime and cement, as separate and as mixtures, increases the UCS values for both curing conditions, marking an improvement in the UCS soil.

Figure 5a illustrates the UCS results of untreated and treated mixtures compared to the native sample and stabilized IPW-lime samples at 28 days in both soaked and unsoaked conditions. The UCS values of the native silty sand sample were 42 kPa and 775 kPa under soaked and unsoaked conditions, respectively. The increasing ratio of IPW by 5% and increasing lime ratio by 2% observed the improvement in the UCS for different mixtures at 28 days for both curing conditions. The Pozzolan reaction between lime and soil particles confirms the idea of bonding between particles of soil once the flocculation process is completed in the first weeks. The mixture of 5% IPW with 6% lime shows the significant increases in UCS, with 1145 kPa and 1505 kPa displaying 27.6 times and 1.94 times increase in UCS after the soil was reinforced under unsoaked and soaked conditions, respectively. The stabilized soil samples P5L2 and P5L4 treated with 5% PET with 2% and 4% lime, respectively, develop a compressive strength of around 640 kPa and 1080 kPa, attesting to a 15.2 times and 25.7 times increase at 28 days for unsoaked conditions. and of around 850 kPa and 1270 kPa, attesting to a 1.1 times and 1.63 times increase at 28 days for soaked conditions, respectively, compared to the UCS of the untreated soil. The mixtures with 5% IPW influence the UCS of samples rather than the addition of 10% of IPW; this can be explained by the decrease in bonding between the soil particles. On the other hand, the UCS value of the stabilized soil sample P5L2C2 by adding 5% IPW strips combined with both 2% lime and 2% cement was 1315 kPa and 1590 kPa under unsoaked and soaked conditions, displaying 31.3 times and 2.05 times increases, respectively. Furthermore, it has improved to 875 kPa and 1200 kPa under unsoaked and soaked conditions for the mixture of P10L2C2 (10% IPW + 2% lime + 2% cement), displaying 20.8 times and 1.54 times increase under unsoaked and soaked conditions. The improvement of UCS values under unsoaked and soaked conditions indicates an improvement in the soil’s strength.

Figure 5b illustrates the UCS values of stabilized mixtures with IPW-lime-cement compared to the native and stabilized IPW-cement samples at 28 days in both curing conditions. For the IPW-cement samples, it has been observed that the addition of 5% IPW shreds and an increase in the ratio of cement by 2% to 6% lead to a significant improvement in the UCS values at 28 days for unsoaked and soaked conditions. While doubling the amount of IPW at 10%, the evolution of UCS values was a slow and close improvement compared to the UCS of samples with 5% IPW.

The great improvement in UCS results was observed when adding 5% IPW with 6% cement, resulting in 1645 kPa and 2130 kPa, displaying 39.1 times and 2.74 times the increase in UCS after the soil was reinforced under unsoaked and soaked conditions, respectively. The P5C2 dry soil samples (5% IPW with 2% cement) and P5C4 (5% IPW with 4% cement) developed a compressive strength of around 950 and 1390 kPa, attesting to 22.6 times and 33.9 times increase for unsoaked conditions, respectively. While for the soaked condition, the improvements were not as great, with around 1370 kPa and 1780 kPa, attesting to a 1.76 times and 2.3 times increase, respectively, compared to that of the native soil.

According to the UCS results of all samples, the UCS values of the soaked condition were always greater than those in the unsoaked condition. But the samples that were in unsoaked conditions performed excellently compared to the UCS of the native sample, while the smallest improvement exceeded 15 times the native soaked UCS value. This indicates that a sample containing 5% IPW has a higher effect on the UCS of the stabilized sample than the 10% IPW content one. This may be related to the smaller amount of binder, which causes more void spaces and gaps between soil particles. As binder particles decrease, the soil sample becomes less dense and less compact. The UCS strength of reinforced soil using IPW combined with binder increases with the increase in both IPW and binder content. These results are in accordance with the literature proposed by [36,51,52]. Increasing UCS results when adding IPW with binder to the soil might be related to the bridging and roughness surface effect of the IPW shreds, which can effectively prevent the future occurrence of failure in the soil. However, comparing both results shows that cement hydrates at a higher rate than lime addition. This effect is logical, considering the slow rate of pozzolan reaction of lime-treated soils, which can take months to years to achieve the desired strength, depending on curing conditions [14]. We conclude that cement gives a good result compared to lime because of the rapid hydration of the cement, a chemical process where water reacts with the cement, leading to faster and stronger binding, more than lime. But we can enhance the effect of using the IPW lime mixture by combining it with a cement amount to increase the efficiency of the combination.

### 3.4. Morphology Analysis

#### 3.4.1. SEM

**Figure 6:**
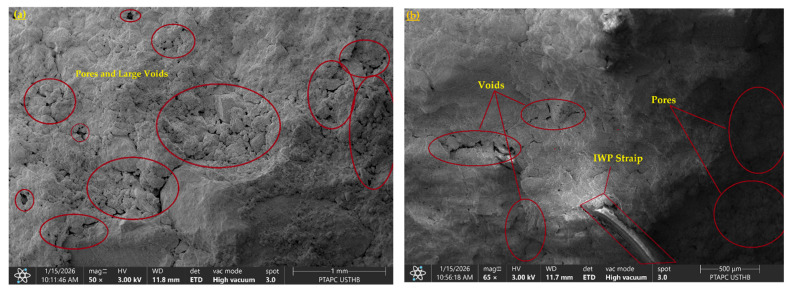

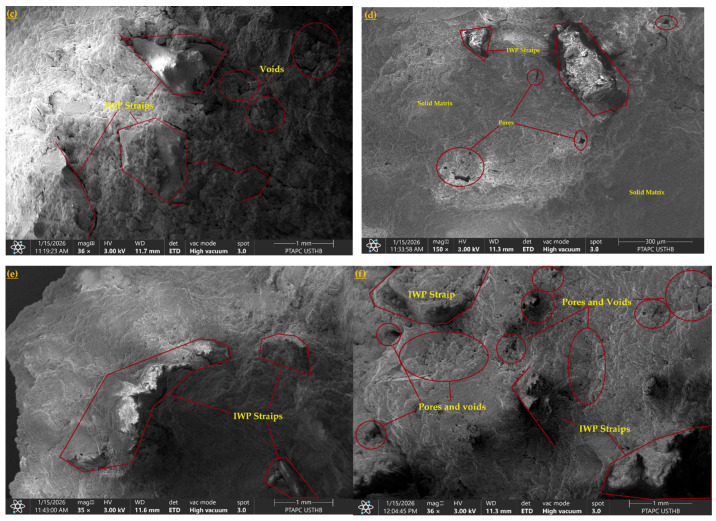
Scanning electron microscopic images SEM of the natural and stabilized soil samples (**a**) Native, (**b**) P5L4, (**c**) P10L4, (**d**) P5C4, (**e**) P5L2C2, and (**f**) P10L2C2.

Figure 6 presents SEM micrographs that provide insights into the stabilization of dispersive soil. The untreated soil (Figure 6a) appears to exhibit a relatively loose structure with noticeable voids and dispersed particles, which may be associated with its low mechanical performance. In contrast, soils treated with hybrid stabilization using industrial waste plastic (IPW) combined with lime and/or cement (Figure 6b–f) show a more compact and organized fabric. These observations may suggest the possible formation of cementitious products such as calcium silicate hydrate (C–S–H) and calcium aluminosilicate hydrate (C–A–S–H), which could contribute to void filling and improved particle bonding. In the presence of IPW shreds, the microstructure appears denser and more uniform, which may indicate improved particle arrangement.

Overall, the combined presence of IPW and cementitious products may contribute to reduced porosity and improved mechanical behavior of the treated soil. The mixtures containing 5% IPW with 4% cement (P5C4), and 5% IPW with 2% lime and 2% cement (P5L2C2), appear to show comparatively more favorable microstructural features (Figure 6d,e), which may be associated with enhanced stabilization. The IPW shreds may increase interparticle friction and provide potential surfaces for the deposition or attachment of cementitious compounds, thereby contributing to improved soil–matrix interaction. As observed in Figure 6b,d,e, cementitious phases appear to be distributed on or around the surfaces of IPW elements, which may contribute to improved structural integrity of the composite. The irregular geometry of IPW shreds may also provide a potential reinforcing effect by redistributing stresses and limiting crack propagation. This behavior may contribute to improved strength and durability, particularly under environmental loading conditions. However, increasing the IPW content to 10% with 4% lime (P10L4, Figure 6c) appears to result in less favorable microstructural characteristics. The images suggest the presence of micro-cracks, localized voids, and possible partial detachment of cementitious products, which may be related to the observed reduction in stiffness, strength, and durability. By contrast, the P5L2C2 and P5C4 specimens (Figure 6e,f) exhibit a relatively more stable microstructure, where IPW elements appear to bridge micro-cracks and help maintain particle connectivity. This observation may partly explain the improved mechanical performance and reduced weight loss observed during wetting–drying durability cycles.

#### 3.4.2. EDS

**Figure 7:**
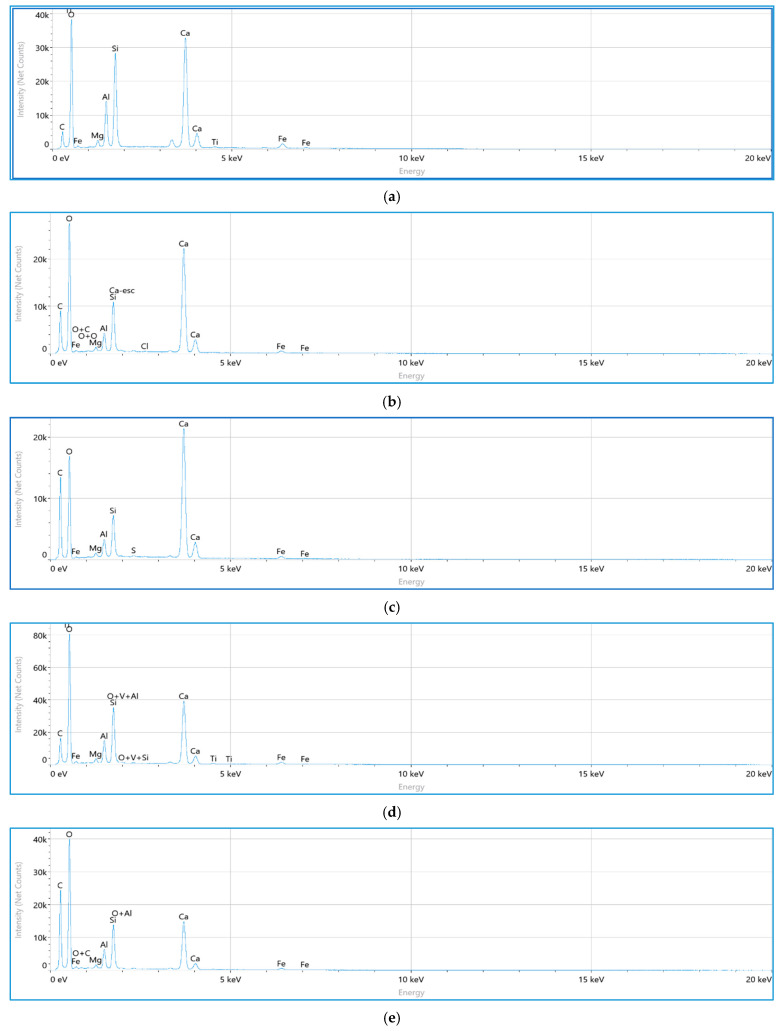

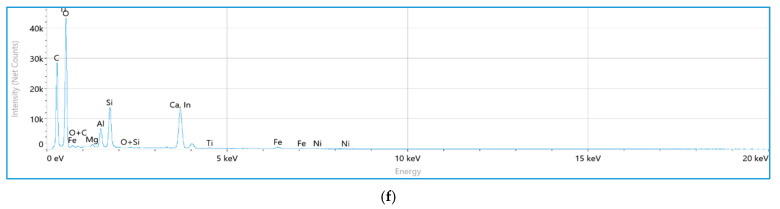
Energy Dispersive Spectroscopy (EDS) patterns of the natural and stabilized soil samples (**a**) Native, (**b**) P5L4, (**c**) P10L4, (**d**) P5C4, (**e**) P5L2C2, and (**f**) P10L2C2.

The results of the EDS analyses for natural and treated dispersive soil samples stabilized with industrial waste plastic (IPW), lime, and cement are presented in Figure 7.

Figure 7a shows the EDS spectrum of the untreated soil, which appears to be primarily composed of silica- and calcium-bearing constituents, as indicated by dominant O, Si, and Ca peaks, along with moderate Al and minor Mg, Fe, and Ti signals. This composition may be consistent with the presence of quartz (SiO_2_) and calcite (CaCO_3_). The absence of Ca–Si ratios typically associated with hydration products suggests that cementitious phases are unlikely to be present in the natural soil, providing a baseline condition for evaluating treatment effects. For lime-treated samples (P5L4 and P10L4; Figure 7b,c), relatively high calcium contents and elevated Ca/Si ratios (≈2.9 and ≈4.2, respectively) are observed. These values may indicate the presence of residual or partially carbonated lime. The detected aluminum (Al/Si ≈ 0.4–0.5) may suggest that some pozzolan reactions could have occurred, potentially leading to the formation of calcium aluminosilicate hydrate-type phases. The higher Ca/Si ratio in P10L4 compared to P5L4 may reflect less efficient calcium consumption, possibly due to reduced contact between lime and reactive soil particles. This behavior may be influenced by the increased IPW content, which could affect particle packing and accessibility rather than directly participating in chemical reactions. In Figure 7d, sample P5C4 exhibits a Ca/Si ratio of approximately 1.56, which is often reported in the literature for hydrated cement systems. This may be indicative of the formation of calcium silicate hydrate (C–S–H)-type phases or related aluminosilicate hydrates. Compared to lime-treated samples, the lower Ca/Si ratio may suggest more effective calcium utilization, consistent with the higher reactivity of cement. For blended mixtures, sample P5L2C2 (Figure 7e) shows a Ca/Si ratio of approximately 1.58, which may indicate relatively balanced reaction conditions and the possible development of calcium aluminosilicate hydrate-type phases. However, the sample shown in Figure 7f presents a higher Ca/Si ratio (~2.95), which may suggest the presence of unreacted or excess calcium. This variation may again be influenced by the higher IPW content, which could affect particle contact and reaction efficiency. Overall, the EDS results suggest that lime-treated soils tend to exhibit higher Ca/Si ratios, which may be associated with residual calcium due to the limited availability of reactive aluminosilicate phases in silty sand. Cement-treated soils appear to show comparatively lower Ca/Si ratios, which may indicate more efficient incorporation of calcium into hydration products. Lime–cement blends may promote more balanced calcium utilization and the possible formation of mixed calcium aluminosilicate hydrate-type phases. Increasing IPW content appears to influence the distribution of reaction products by modifying soil structure, which may slightly affect reaction efficiency in some cases. This behavior could help explain the observed variations in mechanical performance and durability, including reductions in strength and increased weight loss during wetting–drying cycles at higher IPW contents. It is important to note that EDS provides semi-quantitative elemental information, and the interpretation of hydration products such as C–S–H or C–A–S–H is based on inferred elemental trends and typical literature ranges, rather than direct phase identification.

#### 3.4.3. XRD

**Figure 8:**
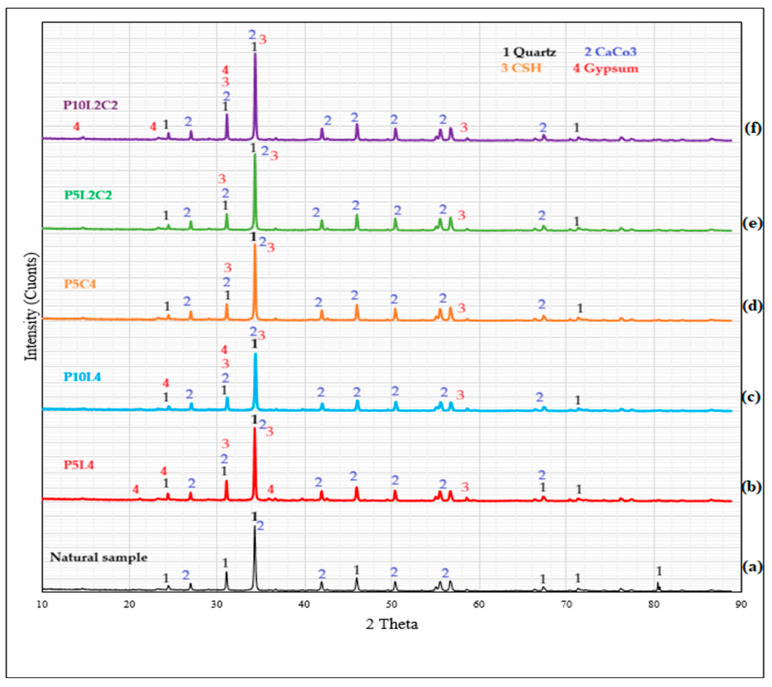
X-ray diffraction (XRD) patterns of the natural and stabilized soil samples (**a**) Native, (**b**) P5L4, (**c**) P10L4, (**d**) P5C4, (**e**) P5L2C2, and (**f**) P10L2C2.

X-ray diffraction (XRD) analysis was conducted to determine the mineralogical composition of the natural sample and five selected stabilized silty sand samples, as presented in Figure 8.

Strong, sharp diffraction peaks corresponding to Quartz were observed in all six samples, confirming Quartz as the dominant component of the natural soil. Secondary peaks associated with Dolomite and calcium carbonate indicate the presence of magnesium–calcium carbonate phases within the soil matrix, contributing to its crystalline structure and potential cementation effects. In the stabilized samples, quartz remained the dominant inert phase. At the same time, new peaks corresponding to Calcium hydroxide and possible Ettringite formation were detected, confirming hydration reactions due to lime and cement addition. A slight increase in amorphous background intensity between 12° and 36° (2θ) suggests the formation of poorly crystalline cementitious products, mainly Calcium silicate hydrate, calcium aluminate hydrate (C-A-H), and weak Calcium aluminosilicate hydrate phases. Owing to the poor crystallinity of C–S–H and C–A–S–H gels, their low peak intensity does not necessarily indicate low concentration; SEM–EDS analysis previously performed confirms their presence and supports the proposed stabilization mechanism.

### 3.5. Effect of the Combination of IPW with Lime and Cement on the Durability

**Figure 9:**
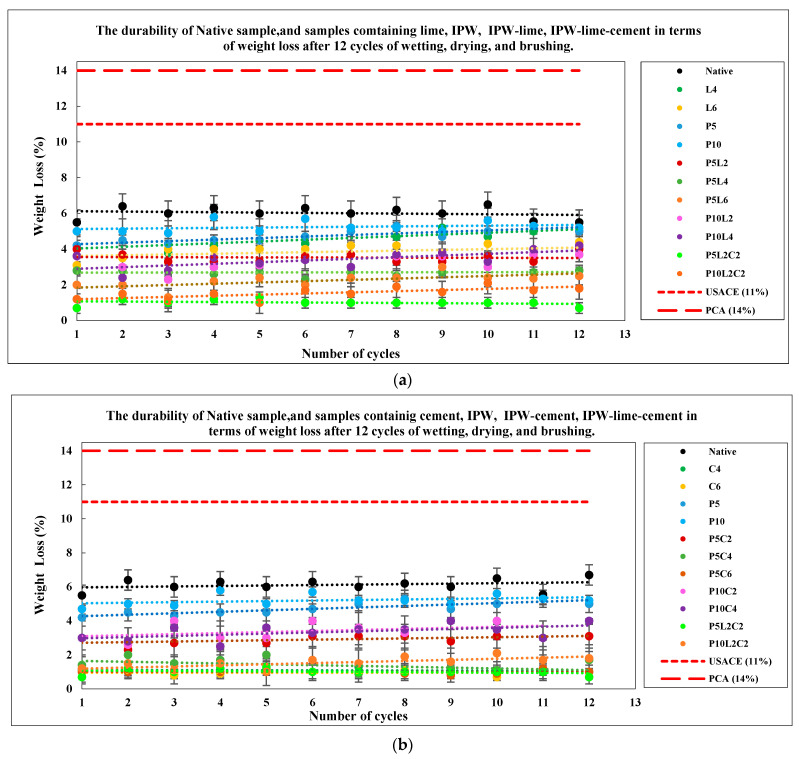
Effect of the combination of IPW with lime and cement on the durability of samples.

Durability tests were conducted in accordance with ASTM D559 (2015) [53] using wetting–drying cycles. A total of nineteen (19) mixtures were evaluated, with three specimens prepared for each mixture as described in Section 2.2. The influence of lime, cement, and IPW—used individually and in combination—on weight loss (WL) was assessed over 12 wetting–drying–brushing cycles. After a curing period of seven days, specimens were oven-dried for at least 48 h, then brushed using a metal bristle brush under a force of approximately 13–16 N, applying 20 strokes uniformly across all faces and edges. The detached material was collected and weighed to determine WL. Each cycle was completed by immersing the specimens in water for 6 h prior to the next sequence. Figure 9 presents the durability performance of treated and untreated samples in terms of WL after 12 cycles.

The durability results in Figure 9a indicate that the natural soil exhibits the highest weight loss (WL ≈ 6%), confirming its low resistance to cyclic degradation. Stabilization improves performance; however, its effectiveness depends on the type and dosage of additives. Samples reinforced with IPW alone (P5 and P10) show behavior comparable to the natural soil, indicating that fibers without adequate bonding have a limited influence on durability. Lime treatment improves early-cycle performance, as observed for L4 (WL reduced to ≈ 4% within the first six cycles), but its effectiveness decreases at later stages. In contrast, L6 and P5L2 maintain a stable WL of about 4%, reflecting improved durability due to enhanced pozzolanic reactions.

A clear synergistic effect is observed when lime is combined with 5% IPW, where increasing lime content (P5L2–P5L6) progressively reduces WL. However, mixtures with 10% IPW (P10L2 and P10L4) exhibit higher losses, suggesting that excessive fiber content weakens the matrix by increasing voids and reducing bonding efficiency. The inclusion of cement further enhances durability, with P5L2C2 showing the lowest WL (≈1%) due to the formation of a dense cementitious matrix. Increasing IPW to 10% slightly increases WL, confirming the negative effect of excessive fiber dosage.

Figure 9b highlights the dominant role of cement, where C4 and C6 reduce WL to approximately 3% and 2%, respectively, with similar improvements observed in P5C4 and P5C6. Overall, cement stabilization is more effective than lime, particularly when combined with an optimal fiber content of 5% IPW. The best performance is achieved by P5L2C2, indicating its suitability for applications subjected to cyclic environmental or mechanical loading, such as subgrades and embankments. Although all samples satisfy the allowable WL limits specified by the Portland Cement Association (14%) and the U.S. Army Corps of Engineers (11%), the results emphasize the importance of optimized mix design for long-term durability.

### 3.6. Effect of the Combination of IPW with Lime and Cement on Resilient Modulus Mr Values

In the mechanical design of pavement layers, the Resilient Modulus (MR) is a crucial parameter that guarantees precise values and a safe and cost-effective design [54]. Mr is an elastic modulus that responds to cyclic loading. In a cyclic triaxial test, repeated deviatoric stress is divided by a specimen’s corresponding recoverable axial strain [48,55]. Furthermore, Mr is the proportion of stress to strain for quickly applied loads—like those experienced by pavements, which measure subgrade material stiffness. In contrast, the modulus of elasticity (E) is a parameter used to measure the material or the resistance of the material to elastic deformation when the load is applied slowly [56]. The AASHTO, 1986 guide [57] recommends using Mr for designing flexible pavements. Mr. is one of the common crucial characteristics of pavement materials since it predicts recoverable stress, strain, and permanent deformation. Several testing techniques have been developed in recent decades to determine the Mr of various pavement materials, including the triaxial test, cyclic plate-loading test, torsional shear test, and so on. A number of correlations were also developed using simple laboratory and field test data, such as the CBR, UCS, and so on, to estimate the Mr values [48,55].

In this study, the resilient modulus (Mr) values for the natural and stabilized silty sand soils were estimated from CBR results using the empirical equation Mr = 62.63 + 0.1582 × ${CBR}^{1.6}$, reported by [48], rather than being obtained through direct resilient modulus testing. The proposed correlation was established for silty soils treated with lime and lime–cement mixtures under controlled laboratory conditions.

Their study demonstrated that CBR-based approaches can provide practical estimates of Mr, particularly for stabilized fine-grained soils used in pavement base layers. Furthermore, Primusz et al. [48] validated their laboratory-derived modulus values through field measurements on an experimental road section, showing reasonable agreement between estimated and measured stiffness values. This supports the applicability of such correlations for preliminary engineering evaluation. However, it is also recognized that CBR–Mr relationships are material-specific and may exhibit variability depending on soil composition, moisture content, and type of stabilization. In particular, the presence of industrial waste plastic in the current study introduces additional uncertainty, as such materials were not considered in the original formulation. Therefore, the estimated Mr values are interpreted as approximate indicators for comparative analysis rather than absolute design parameters. A direct measurement of Mr using cyclic loading tests is recommended for future studies to validate the observed trends.

**Figure 10:**
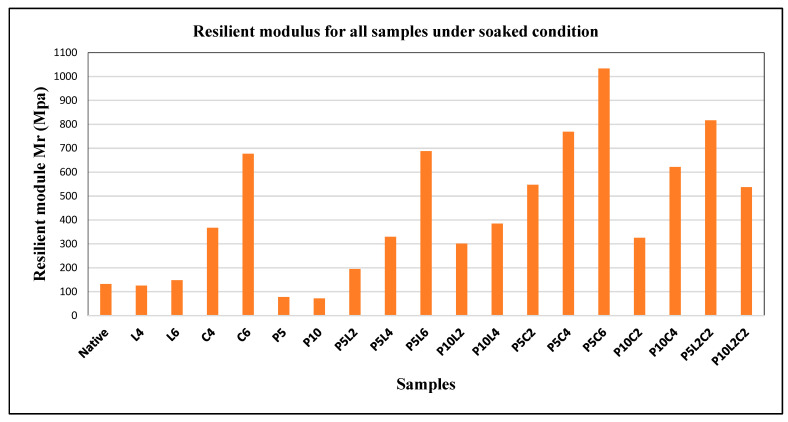
Effect of IPW and binders on Mr for different mixtures in the soaked condition.

The results are presented in Figure 10, which indicates that in the soaked condition (to mimic flooding conditions), the Mr value of the native sample was estimated at 131.8 MPa, for the stabilized samples with lime, cement, and IPW shreds are ranged between 125 and 148 MPa, 367 and 676 MPa, and 72 and 77 MPa, respectively. For stabilized samples with industrial waste plastic (IPW) shreds—combined with lime and cement separately and as a mixture—the values ranged from 195 to 1033 MPa.

Most of the Mr values of these sample results conform to the Mr required for pavements recommended by AASHTO 1993 [58]. Taking into account the Mr value given by the samples (a high of 600 MPa), the effects of combining the IPW shreds with cement (C6, P5L6, P5C4, and P5C6) and IPW with both lime and cement (P5L2C2) are evident for various contents. Confirming significant increases in the stiffness of silty sand soil when mixing 5% of IPW shreds with both stabilization binders or with cement concurrently than separately using IPW shreds with lime or adding 10% of IPW. However, the addition of lime with 4% or 6%, and the addition of 5% or 10% of IPW gave very weak values in global, and this does not match the required values for the subgrade resilient modulus. This investigation’s findings shed light on the potential of industrial waste plastic IPW combined with small amounts of lime and cement as an alternative technique for subgrade soil stabilization. According to the literature, the minimum resilient modulus is about 300 MPa, a threshold that our work surpassed in various mixtures, further underlining the importance of our findings.

## 4. Conclusions

In this study, we approached a wide experimental campaign focused on stabilizing silty sand soil by incorporating industrial waste plastic shreds (IPW) with lime and cement in various percentages. The objective was to evaluate the impact of this combination of additives on the properties of this silty sand soil. The following conclusions and recommendations have been derived from this research:

- The combined addition of IPW with lime and cement separately and as mixtures significantly enhances and stabilizes silty sand soils, resulting in notable improvements.
- Incorporating IPW shreds increases the durability of the cementitious materials used in the stabilization technique.
- Significant improvements in the CBR and UCS values were obtained in both soaked and unsoaked conditions.
- The industrial waste plastic with Portland cement demonstrated high effectiveness in the stabilization of silty sand soil due to the coordination of C-S-H gel with IPW shreds.
- Quicklime contributes to the stabilization of IPW-reinforced soil through the common ion effect and gypsum precipitation mechanisms. However, Portland cement exhibits a more pronounced improvement in mechanical properties compared to quicklime when combined with IPW shreds.
- The combination of 5% of IPW shreds with both 2% quicklime and 2% Portland cement produced a synergistic effect. The combination of these binders provides a balanced enhancement of both environmental and mechanical stability.
- The addition of IPW, lime, and cement improved the strength characteristics of the silty sand soil. The estimated resilient modulus (Mr) values, derived from empirical equations, suggest that the stabilized soil may be suitable for use as subgrade material in flexible pavement construction.
- This study proposes a more effective method for disposing of industrial waste plastic than recycling, landfilling, or dumping it outdoors.

While the results of this study highlight the significant potential of combining industrial waste plastic (IWP) shreds with cementitious materials for improving the engineering properties of degraded soils, additional investigations are still required. The findings demonstrate the importance of innovative and sustainable approaches in civil engineering applications involving problematic soils. However, further research is necessary to evaluate the long-term environmental performance of the proposed stabilization technique, particularly regarding the potential leaching of additives, dyes, residual contaminants, or other pollutants from the industrial plastic waste. In addition, challenges related to large-scale implementation and field performance should be further investigated. It should also be noted that the resilient modulus (Mr) values reported in this study were estimated using an empirical equation rather than obtained through direct laboratory testing. Furthermore, the presented results correspond to the early curing period; therefore, long-term durability and environmental assessments should be considered in future studies.

## Figures and Tables

**Table 1:** Physical Properties of the natural soil sample.

Parameters	Values
Specific gravity, Gs	2.69
Coefficient of uniformity, Cu (%)	45.71
Coefficient of curvature, Cc (%)	1.45
Liquid limit, LL (%)	39.13
Plastic limit, PL (%)	25.88
Plasticity index, PI (%)	13.25
Gravel (%)	1.3
Silt (%)	27.8
Sand (%)	70.9
Maximum dry density, MDD (kN/m^3^)	18.3
Optimum water content, OMC (%)	14
USCS classification	SM
AASHTO classification	A-2-4

**Table 2:** Chemical Components of the natural soil sample.

Constituent	Percentage (wt.%)
${SiO}_{2}$	51.2
CaO	13.7
${Al}_{2}O_{3}$	9.4
$K_{2}O$	1.1
${Fe}_{2}O_{3}$	7.9
MgO	2.7
${TiO}_{2}$	1.6
Other impurities	12.4

**Table 3:** Properties of the quicklime.

Material	Lime (%)
Physical Appearance	Dry white powder
Cao	>83.3
MgO	<0.5
Fe_2_O_3_	<2
Al_2_O_3_	<1.5
SiO_2_	<2.5
SO_3_	<0.5
Na_2_O	0.4–0.5
CO_2_	<5
CaCO_3_	<10
Specific gravity	2
More than 90 μm (%)	<10
More than 630 μm (%)	0
Insoluble material (%)	<1
Apparent density (g/L)	600–900

**Table 4:** Physical properties and the chemical composition of Cement, according to [47].

Physical Properties	Normal Consistency	25–28.5
	Blaine Specific Surface	4160–5270 μm/m
	Start of Setting	135–190 min
	End of Setting	190–285 min
	Shrinkage at 28 Days	<1000 μm/m
	Expansion	0.25–2.55 mm
	Compressive Strength at 28 Days	≥42.5 MPa
Chemical Composition	Loss on ignition	7–12.5%
	Insoluble residues	0.7–2%
	Sulfates SO_3_	2–2.7%
	Magnesium oxide MgO	1–2.2%
	Chloride content	0.01–0.05%
	Tricalcium silicates C_3_S	55–62%
	Alkaline equivalent content	0.5–0.75%

**Table 5:** Compositions of stabilized soils used in laboratory tests.

N		ID	Additives Mixtures
1	Natural Soil	Native	No additives
2	Lime-treated soil	L4	4% Lime
3		L6	6% Lime
4	Cement-treated soil	C4	4% Cement
5		C6	6% Cement
6	Industrial Waste Plastic-treated soil	P5	5% IPW
7		P10	10% IPW
8	Hybrid-treated soil by IPW with Lime	P5L2	5% IPW + 2% lime
9		P5L4	5% IPW + 4% lime
10		P5L6	5% IPW + 6% lime
11		P10L2	10% IPW + 2% lime
12		P10L4	10% IPW + 4% lime
13	Hybrid-treated soil by IPW with Cement	P5C2	5% IPW + 2% cement
14		P5C4	5% IPW + 4% cement
15		P5C6	5% IPW + 6% cement
16		P10C2	10% IPW + 2% cement
17		P10C4	10% IPW + 4% cement
18	Hybrid-treated soil by IPW with Lime and Cement	P5L2C2	5% IPW + 2% lime + 2% cement
19		P10L2C2	10% IPW + 2% lime + 2% cement

## Data Availability

The original contributions presented in the study are included in the article, further inquiries can be directed to the corresponding author.
